# High-throughput physical vapour deposition flexible thermoelectric generators

**DOI:** 10.1038/s41598-019-41000-y

**Published:** 2019-03-13

**Authors:** Katrina A. Morgan, Tian Tang, Ioannis Zeimpekis, Andrea Ravagli, Chris Craig, Jin Yao, Zhuo Feng, Dmitry Yarmolich, Clara Barker, Hazel Assender, Daniel W. Hewak

**Affiliations:** 1 Optoelectronics Research Centre, University of Southampton, Southampton, UK; 2 Department of Materials, University of Oxford, Oxford, UK; 3 Plasma App ltd., Rutherford Appleton Laboratory, Harwell Oxford Science and Innovation Campus, Building R18 Fermi Avenue, Didcot, UK

## Abstract

Flexible thermoelectric generators (TEGs) can provide uninterrupted, green energy from body-heat, overcoming bulky battery configurations that limit the wearable-technologies market today. High-throughput production of flexible TEGs is currently dominated by printing techniques, limiting material choices and performance. This work investigates the compatibility of physical vapour deposition (PVD) techniques with a flexible commercial process, roll-to-roll (R2R), for thermoelectric applications. We demonstrate, on a flexible polyimide substrate, a sputtered Bi_2_Te_3_/GeTe TEG with Seebeck coefficient (S) of 140 μV/K per pair and output power (P) of 0.4 nW per pair for a 20 °C temperature difference. For the first time, thermoelectric properties of R2R sputtered Bi_2_Te_3_ films are reported and we demonstrate the ability to tune the power factor by lowering run times, lending itself to a high-speed low-cost process. To further illustrate this high-rate PVD/R2R compatibility, we fabricate a TEG using Virtual Cathode Deposition (VCD), a novel high deposition rate PVD tool, for the first time. This Bi_2_Te_3_/Bi_0.5_Sb_1.5_Te_3_ TEG exhibits S = 250 μV/K per pair and P = 0.2 nW per pair for a 20 °C temperature difference.

## Introduction

Thermoelectric generators (TEGs) can provide constant power for flexible electronic platforms. Using the body’s warmth, they do not rely on solar power, unlike photovoltaic generators, or on the user’s fitness, unlike electromagnetic induction generators. TEGs could be combined with sensors and displays to enable a fully flexible integrated circuit to achieve commercialisation viability^1^. However there are still challenges that are holding back this technology from fully entering the market. These include limited efficiencies at body range temperatures, large-area scaling and mass production compatibility^2–4^.

The efficiency of thermoelectric materials are measured by a unitless value known as the figure of merit, ZT, defined by Eq. (1), where σ, S, T and κ are the electrical conductivity, Seebeck coefficient, temperature and thermal conductivity, respectively. In order to increase ZT, thermal conductivities have been lowered by harnessing 2D and nano-structured material properties^5,6^. However the majority require complex fabrication techniques that are extremely challenging to perform on a large-area, mass production scale^7,8^. Alternatively, the efficiency can be raised using scalable techniques by increasing the electrical conductivity and Seebeck coefficient, collectively known as the power factor (PF), which are linked to the materials’ physical properties and defined by Eq. (2). This can be done by discovering new materials or optimising existing ones through techniques such as alloying or doping, where doping can also be used to tune the semiconductor type^9–11^.

1 $$ZT=\frac{\sigma {S}^{2}T}{\kappa }$$

2 $$PF=\sigma {S}^{2}$$

In the commercial world, roll-to-roll (R2R) systems are used to create large areas of high-throughput flexible coatings, and can be used to manufacture flexible electronics. Rolls of flexible materials, referred to as a web, are gradually unwound, coated and rewound, with the coated processes potentially consisting of multiple steps/layers. Web speeds may be, for example, up to hundreds of metres per minute, allowing a high-throughput manufacturing process. Inkjet printing is a well-known technique that is scalable and compatible with R2R but has many limiting factors; the ink must maintain low surface tension, low viscosity, and have the nanomaterial well dispersed^12^. This creates challenges with synthesizing inks, limiting material choices, and can lead to non-uniform films with poor density and electrical conductivity, limiting power factors^13^. Sputtering, a type of PVD, is an alternative, scalable and R2R compatible technique that offers high quality films from a huge array of materials with the ability to tightly control material properties, enabling PFs of materials to be tuned^14,15^.

To date, published research reporting on sputtered flexible TEGs is limited, often showing only small-scale one off prototypes or requires complex photolithography or post-processing steps, making it less attractive for use in an R2R environment^16–24^. Alternatively, papers are focused on the material itself and do not take into account the up-scaling of flexible generators^25–34^.

In this work we investigate the suitability and potential of using PVD techniques with R2R for high-throughput manufacturing of flexible TEGs. We demonstrate sputtering as a viable technique for producing a flexible TEG, first by screening materials (section i) and then by selecting two materials to produce a flexible TEG prototype (section ii). To investigate PVD/R2R compatibility for thermoelectric applications, we investigate thermoelectric properties of R2R sputtered Bi_2_Te_3_ for the first time (section iii). Further to this, we highlight that higher PFs can in fact be tuned by reducing deposition times, demonstrating PVD/R2R’s potential as a high-speed, low-cost commercial system for flexible electronics. Identifying faster deposition times as beneficial, we use a novel high-deposition rate PVD technique, virtual cathode deposition (VCD), to make a TEG prototype for the first time (section iv). VCD offers deposition speeds of more than 1 µm/min whilst maintaining substrate temperatures to below 60 °C, making it fully compatible with a large array of low temperature flexible substrates and has potential to be the future of high-throughput flexible electronic manufacturing via R2R.

## Results and Discussion

### Sputtered Te thin films

In order to identify possible materials with high PFs, an equation estimating an optimal bandgap of a material, based on the operating temperature of the device (T) can be used, given by Eq. (3), where k_B_ is the Boltzmann constant^35^. For the body temperature regime the estimated band gap is E_g_ = 0.26 eV. Therefore Bi_2_Te_3_ (E_g_ = 0.2 eV), SnTe (E_g_ = 0.2 eV), and GeTe (E_g_ = 0.1–0.8 eV, depending on crystallinity), which exhibit energy gaps close to this optimum, show promise for wearable applications^36–38^. Bi_2_Te_3_ has been widely used as a near room temperature n-type thermoelectric, due to its high ZT of above 1. SnTe has shown promise as a p-type thermoelectric material and more recently GeTe has gained attention as another high performance p-type material, with superior performance attributed to its electronic structure^39^.

3 $${E}_{g}=10\,{k}_{B}T$$

Our preliminary analysis of sputtered Bi_2_Te_3_, SnTe and GeTe films is by EDX, shown in Table 1. All samples are slightly Te deficient compared to the target stoichiometries, with Bi_2.2_Te_2.8_, Ge_1.1_Te_0.9_, Sn_1.1_Te_0.9_. This could be attributed to the significantly higher deposition rate of elemental tellurium compared to elemental germanium or tin, leading to reduced tellurium content of the previously used sputter targets, potentially leading to reduced tellurium when subsequent depositions are performed. The power factor measured on these films are also provided in Table 1. Both n- and p-types are required for TEGs, and as chalcogenides are naturally p-type, it is beneficial to identify a high performance n-type material as the range of n-type chalcogenide materials that are available are more restricted. In these results, both n-type and p-type materials are identified through output voltage vs. temperature plots with the Seebeck coefficient originating from the magnitude of the gradient, and the type originating from the sign of the gradient (see methods section for more detail).

**Table 1:** Atomic concentration of elements and thermoelectric properties of sputtered Bi_2_Te_3_, SnTe and GeTe thin films.

Target Material	Film Compositional Results				Film Electrical Results			
	Bi %	Sn %	Ge %	Te %	Seebeck Coefficient (μV/K)	E Resistivity (mΩ-cm)	Power Factor (μW/cm-K^2^)	Semi-conductor type
Bi_2_Te_3_	43 ± 2	—	—	57 ± 2	−50.6 ± 1.0	0.50 ± 0.05	5.1 ± 0.6	n
SnTe	—	53 ± 2	—	47 ± 2	26.0 ± 1.0	0.20 ± 0.05	3.4 ± 0.9	p
GeTe	—	—	54 ± 2	46 ± 2	47.9 ± 1.0	0.27 ± 0.05	8.5 ± 1.6	p

Error bars represent uncertainty of measurement for EDX, Seebeck coefficient and resistivity. The power factor uncertainty is calculated by error propagation.

The Bi_2.2_Te_2.8_ film exhibits a PF of ~5 μW/cm-K^2^ which is in the expected range based on literature for thin film Bi_2_Te_3_ (3–49 μW/cm-K^2^ varying with thin film deposition technique and deposition parameters)^40–43^. The Sn_1.1_Te_0.9_ film exhibits a PF of ~3 μW/cm-K^2^ which is similar to thermally evaporated SnTe thin films previously published^44^. The Ge_1.1_Te_0.9_ thin film exhibits a *PF* of ~9 μW/cm-K^2^ which is slightly lower than previously reported values, but this could be related to differences in thickness, crystallinity or stoichiometry’s, where 1.7 μm and 1.1 μm films exhibit 16 and 23 μW/cm-K^2^ ^45,46^. The results here mirror similar results seen for bulk GeTe and SnTe materials, where the GeTe outperforms SnTe demonstrating GeTe’s potential for a promising thin film thermoelectric material^39^.

### Flexible Bi_2_Te_3_, GeTe thermoelectric cell

**Figure 1:**
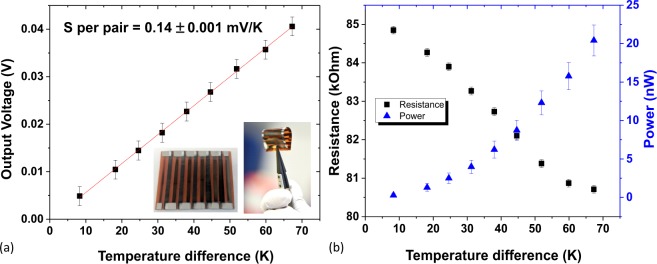
Electrical results for 4.5 pairs of sputtered Bi_2_Te_3_/GeTe flexible TEG (**a**) output voltage vs. temperature difference (error bars of ±2 mV represent uncertainty of voltage reading for this range). (**b**) Measured resistance and calculated power output vs. temperature difference (error bars of ±0.1 kΩ represent uncertainty in resistance reading for this range. The power error bars are calculated through error propagation). Inset in (**a**) is a photo of the device (right hand photo taken by Martyn Roberts, University of Southampton).

Based on section i results, Bi_2_Te_3_ (n-type) and GeTe (p-type) were selected to demonstrate a working flexible sputtered TEG, depicted in the insert of Fig. 1a. For proof of concept, we fabricated five pairs of strips, however power density can be increased by altering the geometry allowing for a higher density of thermoelectric strips (1 strip = 1 n-type and 1 p-type in series).

The voltage output can be seen in Fig. 1a for 4.5 thermoelectric pairs of Bi_2_Te_3_/GeTe. The Seebeck coefficient per pair is extracted as 140 ± 1 μV/K which is larger than previously reported sputtered flexible Bi_2_Te_3_/Sb_2_Te_3_ TEG^18^. As output power is important for TEGs, the resistance of our Bi_2_Te_3_/GeTe cell was measured simultaneous to the output voltage and from this, the output power (assuming Ohmic behaviour) was calculated, as seen in Fig. 1b. Our Bi_2_Te_3_/GeTe TEG exhibits ~0.4 nW per pair at a temperature difference of 20 °C which exceeds the 0.07 nW per pair achieved by the sputtered Bi_2_Te_3_/Sb_2_Te_3_ TEG^18^. The PF and output power can be further improved as sputtering lends itself well to material optimization via co-sputtering (heavy doping), with high control of elemental ratios, and via temperature and pressure settings, enabling control of crystallinity and density^47^. Elemental doping of these sputtered films could also be achieved through ion implantation, which has in some cases shown increased power factor, decreased thermal conductivity or  led to carrier-type reversal, allowing semiconductor types to be tuned^9,11^.

The successful operation of this flexible TEG based on Bi_2_Te_3_ and GeTe demonstrates the proof of concept for using sputtered thin films as a future choice for manufacturing wearable power generation. Even prior to material and geometry optimisation, the GeTe based thermoelectric cells shown in this work demonstrate Seebeck coefficients which exceed sputtered flexible thermoelectric cells based on more traditional materials^18,23,48^. Material properties are highly controllable via sputtering and therefore with further material optimisation and a larger choice of materials than compared with inkjet printing, sputtered TEGs have the ability to outperform printed generators.

### Moving web depositions

To demonstrate large-area high-throughput deposition using PVD and R2R, the performance of Bi_2_Te_3_ films sputtered at room temperature on a rapidly moving polymer substrate is investigated. In order to tune material properties with deposition time, the influence of deposition time on the surface morphology, crystal structure and thermoelectric power factor of the films are characterized, with results shown in Table 2.

**Table 2:** Material and thermoelectric properties for R2R sputtered Bi_2_Te_3_ for different total run times.

Run	Run time (mins)	Thickness (nm)	Average thickness per one rotation (nm/rotation)	S_a_ (μm)	S_q_ (μm)	Seebeck Coefficient (μV/K)	Resistivity (mΩ-cm)	Power factor (μW/cm-K^2^)
1	60	1500 ± 5	1.80 ± 0.01	0.072 ± 0.01	0.108 ± 0.01	−14.1 ± 1.0	1.18 ± 0.05	0.17 ± 0.02
2	30	700 ± 5	1.68 ± 0.01	0.074 ± 0.01	0.110 ± 0.01	−17.2 ± 1.0	2.01 ± 0.05	0.15 ± 0.02
3	15	350 ± 5	1.68 ± 0.02	0.077 ± 0.01	0.110 ± 0.01	−16.8 ± 1.0	1.21 ± 0.05	0.23 ± 0.03
4	5	100 ± 5	1.44 ± 0.07	0.066 ± 0.01	0.097 ± 0.01	−15.7 ± 1.0	1.10 ± 0.05	0.22 ± 0.03

Error bars represent uncertainty of measurements for thickness (profilometry), surface roughness (confocal microscopy), Seebeck coefficient and resistivity. The average thickness per one rotation and the power factor error is calculated through error propagation.

The quality of deposited films via R2R are often evaluated by two properties; thickness and surface roughness^49^. The effect of run time on thickness was investigated. The average thickness per one drum rotation increases with increasing total run time, indicating that the deposition rate increases with time. This is potentially due to Bi_2_Te_3_ adhering better to a seed layer of Bi_2_Te_3_, as opposed to directly onto a blank substrate. The second quality metric for R2R, surface roughness, was found to be independent of run time where average surface roughness, *S*_*a*_, and the root mean square roughness, *S*_*q*_, are shown in Table 2. Whilst the surface roughness is high, this was attributed to the high roughness of the polyethylene terephthalate (PET) substrate itself (*S*_*q,PET*_ = 0.055 um). The surface roughness can be controlled by substrate film selection or by depositing a polymer smoothing layer before sputtering the thermoelectric films^50^.

**Figure 2:**
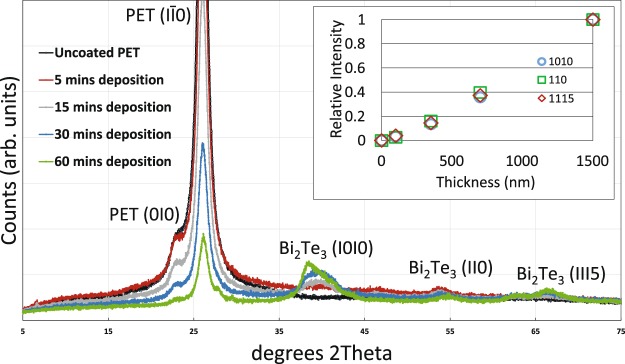
X-ray diffraction patterns of (**a**) pure PET substrate and Bi_2_Te_3_ films on PET substrate grown for (**b**) 5 mins, (**c**) 15 mins (**d**) 30 mins and (**e**) 60 mins. The inset shows relative intensity of each of the bismuth telluride XRD peaks (1010), (110) and (1115), normalised to the most intense PET substrate peak (PET 100) for each of the samples of different thickness.

In order to investigate how run time affects run crystallinity of the films, XRD was performed. XRD traces indicating rhombohedral Bi_2_Te_3_ are in agreement with Zhou’s study and shown in Fig. 2 for different run times^15^.

A plot of the relative intensity of three Bi_2_Te_3_ peaks is shown in the insert in Fig. 2. The relative intensity is calculated by dividing the diffraction peak intensity of the Bi_2_Te_3_ phase by the strongest peak intensity of the PET substrate, after subtraction of the background. All three Bi_2_Te_3_ peaks follow the same trend which is close to linear, as would be expected with increasing thickness, but at low thicknesses the intensity is sub-linear, pointing to the conclusion that the early stages of growth may be less favourable for crystal growth. Opportunely, these potentially less crystalline thin films (run 3 and 4) actually exhibit lower electrical resistivity, as shown in Table 2. This therefore indicates that despite the more amorphous nature of the thinner films, ideal lower electrical resistivity can be achieved through shorter run times, making it even more attractive for high-speed R2R manufacturing.

All roll-to-roll sputtered films demonstrated n-type conduction, as expected based on results from section i. The 30 minute deposition time resulted the highest Seebeck coefficient but suffered from the highest resistivity, leading the lowest power factor. The 15 minute deposition resulted in the highest power factor (0.23 μW/cm-K^2^) but the 5 minute run resulted in a very similar PF (0.22 μW/cm-K^2^). This indicates that the highest power factors can be tuned by lowering the run time, lending itself well to the high throughput R2R needed for commercialisation.

### VCD: a novel high-deposition rate technique

**Figure 3:**
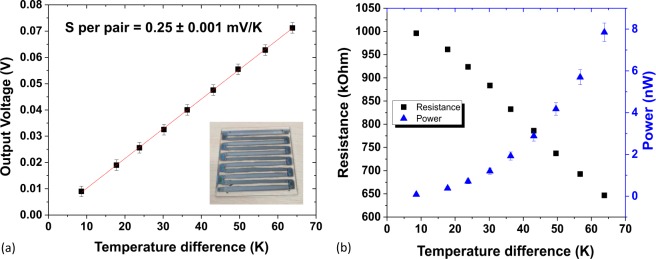
Electrical results for 4.5 pairs of virtual cathode deposited Bi_2_Te_3_/Bi_0.5_Sb_1.5_Te_3_ TEG (**a**) output voltage vs. temperature difference (error bars of ±2 mV represent uncertainty of voltage reading for this range). (**b**) Measured resistance and calculated power output vs. temperature difference (error bars of ±1 kΩ represent uncertainty in resistance reading for this range. The power error bars are calculated through error propagation). Inset in (**a**) is a photo of the device.

In order to demonstrate TEG compatibility with high-rate PVD techniques, high-deposition rate VCD is used to make a proof-of-principle TEG, based on traditional materials Bi_2_Te_3_ and Bi_0.5_Sb_1.5_Te_3_^51^. TEG properties are shown in Fig. 3. This VCD TEG demonstrates S = 250 ± 1 μV/K per pair and ~0.2 nW per pair at a temperature difference of 20 °C, outperforming the sputtered Bi_2_Te_3_/Sb_2_Te_3_ generator^18^. These preliminary VCD generator results offer competitive Seebeeck coefficients, demonstrating VCD’s capacity as a future deposition technique for high-throughput thermoelectric materials.

## Conclusion

PVD is demonstrated here as a R2R compatible process for flexible TEGs, with a wider range of high-quality tuneable materials when compared to traditional R2R printing techniques. A proof-of-concept Bi_2_Te_3_/GeTe TEG made on a flexible polyimide substrate was demonstrated, exhibiting *S* per pair of 140 μV/K and 2 nW of output power for 4.5 pairs, at a temperature difference of 20 °C. The output power can be further improved by reduction of the generator’s internal and contact resistance by altering sputtering conditions. Sputtered Bi_2_Te_3_ thermoelectric properties were tuned for the first time on a R2R system, with desired low electrical resistivity and high PFs achieved by reducing deposition times. This trend lends itself well to R2R manufacturing where speed is key and shorter sputtering times are ideal. As speed is vital in R2R, a novel high deposition rate (>1 μm/min) PVD technique, virtual cathode deposition (VCD), was used to demonstrate a proof-of-concept Bi_2_Te_3_/Bi_0.5_Sb_1.5_Te_3_ TEG prototype, which exhibited *S* per pair of 250 μV/K and an output power of 1 nW for 4.5 pairs at a temperature difference of 20 °C. Combining this novel VCD technique with R2R has potential to take flexible TEGs one step closer to mass production required for the wearable market.

## Methods

### Power Factor Measurements

**Figure 4:**
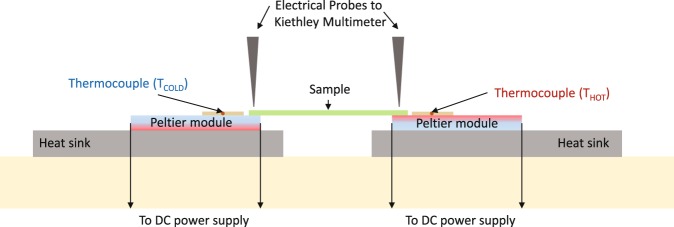
A schematic showing the Seebeck measurement set up used in this work. Two Peltier modules are connected to a DC power supply (one has the hot side face up and one has the cold side face up). The sample is placed on top, with electrical probes applied to the sample to record the output voltage. The temperature of the hot side is increased by increasing the power to the hot-side up Peltier module. Two type K thermocouples are secured on the Peltier modules to measure the temperature difference.

Power factors were calculated from electrical conductivity, measured with Nanometrics HL5550 LN2 Hall system and an in-house Seebeck coefficient setup. A schematic showing the in-house Seebeck measurement set up used in this work is shown in Fig. 4. Two Peltier modules are connected to a DC power supply (one has the hot side face up and one has the cold side face up). The sample is placed on top, with electrical probes applied to the sample to record the output voltage using a Keysight 34410A multimeter. The temperature of the hot side is increased by increasing the power to the hot-side up Peltier module. Two type K thermocouples are secured on the Peltier modules to measure the temperature difference. The output voltage is plotted against the temperature difference, and the magnitude defines the Seebeck coefficient whilst the gradient sign (i.e. positive or negative) indicates the semiconductor type of the film.

### Bi_2_Te_3_, GeTe and SnTe Sputtered Thin Films

A Kurt Lesker Nano38 RF sputterer was used to deposit films on soda lime substrates using Bi_2_Te_3_, GeTe and SnTe sputtering targets at 250 °C chuck temperature, 10^−3^ Torr, with 60W, 45W, 60W and 20 sccm, 37 sccm, 20 sccm of Ar, respectively. The thickness of the Bi_2_Te_3_, GeTe and SnTe thin films were 650 nm, 560 nm and 600 nm ± 5 nm respectively, measured using a KLA-Tencor stylus profiler. A Zeiss Evo50 SEM fitted with an Oxford Instruments INCA 250 x-ray analysis system was used to analyse the composition of the samples with an error of approximately 2%.

### Sputtered Bi_2_Te_3_/GeTe TEG

The parameters and techniques used for the generators are the same as used for the thin films. A polyimide substrate commonly used for thermoelectric flexible devices (Kapton® 500HN 127 um thick) was selected as it has high thermal stability and low thermal conductivity and expansion coefficient for a large array of temperatures^27^. The polyimide was pre-cleaned by soaking it in methanol for 5 minutes followed by a methanol wash, N_2_ dry and baked at 90 °C for 1 hour. Bi_2_Te_3_ was sputtered through a polyimide hard shadow mask with the mask created by laser cutting using a Hobarts Universal PLS6MW Platform. Following deposition of the first half of the strips (the Bi_2_Te_3_), the shadow mask was then moved laterally and GeTe was sputtered. 50 nm of SiO_2_ was then sputtered through a different polyimide hard mask, acting as a capping layer. Finally W/Ag contacts were sputtered through a final polyimide hard mask, where tungsten acts as a diffusion barrier to stop silver diffusion into the thermoelectric strips below^52^.

### Bi_2_Te_3_ Roll-to-roll sputtered films

University of Oxford’s semi-industrial scale R2R vacuum webcoater (Aerre Machines) was used to sputter Bi_2_Te_3_ with 0.25 kW and drum speed of 25 m/min. A low-cost, flexible 12 µm thick PET polymer, commonly used in R2R was used as the substrate. Characterisations were carried out using confocal white light microscopy for surface roughness (Nanofocus AG μsurf), XRD for film crystallization (PANalytical Empyrean Alpha-1 configured with a unique symmetric Ge monochromator (Johansson type) giving Cu Kα1-only Bragg-Brentano reflection geometry data for structure determination), and stylus profilometry for thickness measurements.

### R2R vacuum webcoater setup

**Figure 5:**
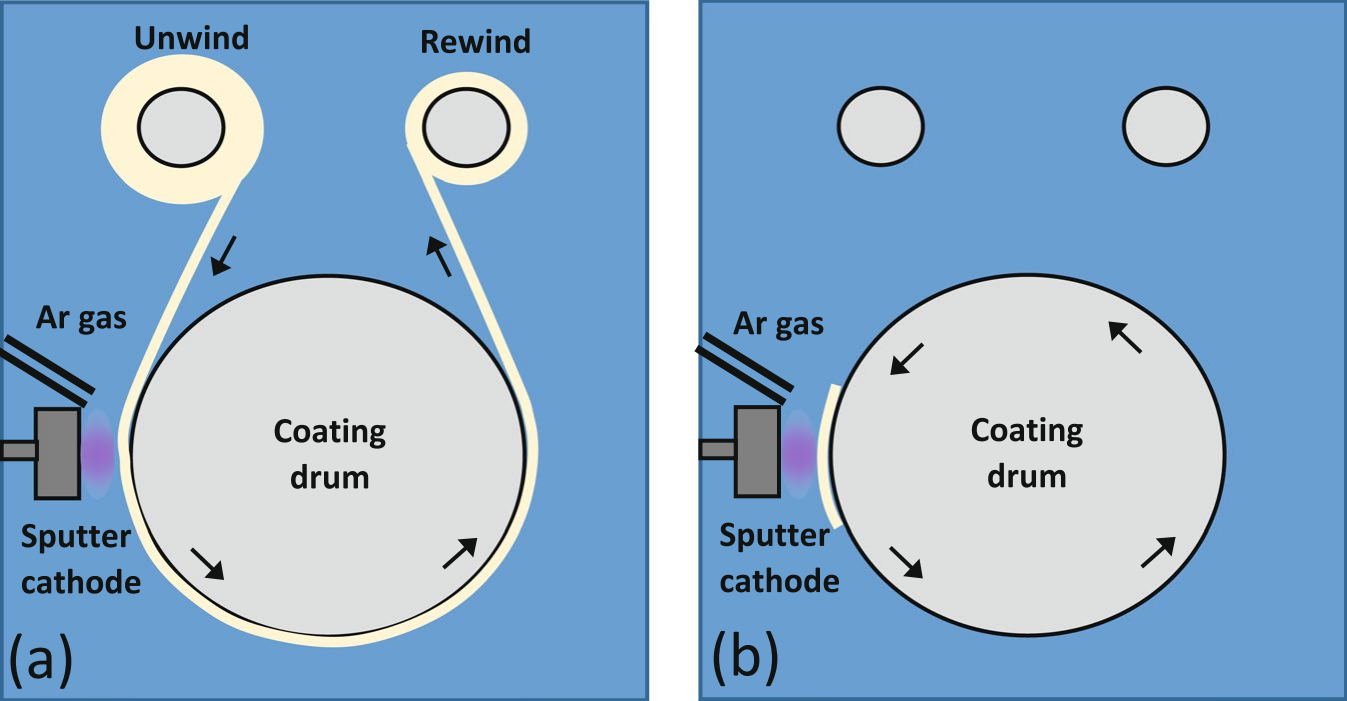
Schematics of roll-to-roll setups. (**a**) R2R setup used in industry with the flexible substrate being unwound from one spool, passing through the deposition area, and then re-wound onto another spool. (**b**) The set up used in this work where the flexible substrate is attached to the coating drum allowing investigations into deposition thickness to be performed.

The R2R setup commonly used in industry was altered for this work to enable a wide range of film thicknesses to be studied, whilst using a single, small, target. Rather than having a continuous roll of substrate, being unwound from one spool, through the sputtering area once, and re-wound onto another spool (Fig. 5a), we attached our substrates directly onto the coating drum, which then passed through the sputtering area multiple times, building up film thickness with every pass (Fig. 5b). Thus we were able to monitor the effect of film thickness without modifying the process conditions. As the target is small compared to the coating drum dimensions (coating drum is 1.8 m in circumference and 0.35 m in width and the DC Bi_2_Te_3_ target is 3 inches), the area in which sputtering occurs will be confined to a relatively small zone of the total coating drum surface. As the drum rotates, there will be large amounts of time where the substrate passes around with the drum, outside the sputter zone, receiving no further build up in thickness. Therefore the actual time of sputtered deposition will be a small fraction of the overall run time (approximately 4% of the drum rotation time being directly in line with the target).

### Bi_2_Te_3_/Bi_0.5_Sb_1.5_Te_3_ VCD TEG

**Figure 6:**
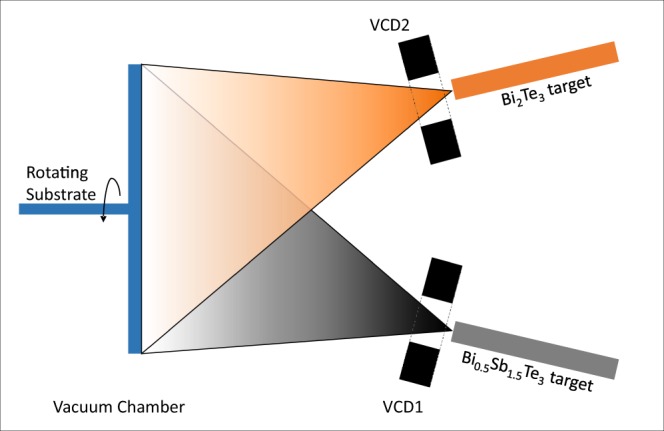
A schematic of the Virtual Cathode Deposition technique used to fabricate the Bi_2_Te_3_/Bi_0.5_Sb_1.5_Te_3_ thermoelectric cell reported in this work. Two identical VCD sources independently driven by two pulsed power sources with computer control (not shown in the schematics) generate plasma plumes of target materials. The plumes direction of expansion and density decrease due to the expansion are schematically demonstrated by the collating.

The same masking method as described for the Bi_2_Te_3_/GeTe TEG was used for the VCD TEG. 1 μm of Bi_2_Te_3_ and Bi_0.5_Sb_1.5_Te_3_ were deposited via VCD onto a borosilicate substrate using 40000 pulses, 2 J per pulse, at 50 Hz with 10 sccm of Ar. The substrate temperature was kept below 60 °C. Following this, 50 nm of SiO_2_ capping layer and W/Al contacts were sputtered using a Kurt Lesker Nano38 RF tool. A schematic of the deposition can be seen in Fig. 6.

### VCD technique

VCD uses a pulsed electron beam to ablate a solid-state target and belongs to the physical vapour deposition techniques that operate in a mid-range vacuum of 10^−3^–10^−4^ mBar^51^. The pulsed electron beam deposition technique is a well-known deposition technique that most often utilises a channel-spark discharge (CSD) for the generation of pulsed electron beams with fluence of 10^8^ W/cm^2^ at the target surface^53,54^. CSD based deposition tools were used for deposition of different complex-stoichiometry materials in the laboratory-scale, however industrial application of CSD are limited by the short lifetime of the cathode^55^. VCD utilizes a virtual plasma cathode which is generated prior to each pulse of the electron beam. Plasma is generated by ionization of an operational gas that becomes the plasma cathode when it acquires a negative high voltage potential with respect to a target, due to the application to the plasma from a high-voltage pulse. The electron beam acquires energy in the potential difference between plasma boundary and the target. The electron beam ablates the target and then the plasma cathode decays, leaving a space for ablated target material in the form of a plasma plume to propagate toward a substrate, where it forms a film. Repetition of the pulse with the rate in the range of 1–600 Hz, which starts with the formation of a new virtual plasma cathode and ends with the depositing of the target material on a substrate, allows a film to grow on the substrate with controlled growth rate and properties.

### Error Analysis

Experimental errors are given by error bars and ± values throughout the paper^56^. The error associated with the Seebeck coefficient and electrical conductivity is ±1 μV/K and 0.05 mΩ-cm, respectively. The errors for power factors are calculated from error propagation of these two uncertainties. The EDX error is approximately 2%. The error for output voltage reading is estimated to be ±2 mV. The error on the resistance is dependent on the tool range used to measure with ±1 kΩ for hundreds of kΩ range, and ±0.1 kΩ for tens of kΩ range. The output power of the generators are calculated by the propagation of these uncertainties. The thickness has an error estimated at 5 nm, whilst the surface roughness error is estimated at 10 nm. The average thickness per one rotation error is calculated by the propagation of thickness error.

## Data Availability

All data supporting this study are openly available from the University of Southampton repository at 10.5258/SOTON/D0697.
